# 1MeTIQ and olanzapine, despite their neurochemical impact, did not ameliorate performance in fear conditioning and social interaction tests in an MK-801 rat model of schizophrenia

**DOI:** 10.1007/s43440-020-00209-9

**Published:** 2021-01-06

**Authors:** Magdalena Białoń, Agnieszka Chocyk, Iwona Majcher-Maślanka, Marcelina Żarnowska, Krzysztof Michalski, Lucyna Antkiewicz-Michaluk, Agnieszka Wąsik

**Affiliations:** 1 Department of Neurochemistry, Maj Institute of Pharmacology, Polish Academy of Sciences, Cracow, Poland; 2 Laboratory of Pharmacology and Brain Biostructure, Department of Pharmacology, Maj Institute of Pharmacology, Polish Academy of Sciences, Cracow, Poland

**Keywords:** 1-Methyl-1,2,3,4-tetrahydroisoquinoline (1MeTIQ), Olanzapine, MK-801, Neurochemical studies, Contextual fear conditioning, Social interaction test (SIT)

## Abstract

**Background:**

The aim of the present study was to evaluate the effect of 1MeTIQ on fear memory and social interaction in an MK-801-induced model of schizophrenia. The results obtained after administration of 1MeTIQ were compared with those obtained with olanzapine, an antipsychotic drug.

**Methods:**

Sprague–Dawley rats received a single injection of MK-801 to induce behavioral disorders. 1MeTIQ was given either acutely in a single dose or chronically for 7 consecutive days. Olanzapine was administered once. In groups receiving combined treatments, 1MeTIQ or olanzapine was administered 20 min before MK-801 injection. Contextual fear conditioning was used to assess disturbances in fear memory (FM), and the sociability of the rats was measured in the social interaction test (SIT). Biochemical analysis was carried out to evaluate monoamine levels in selected brain structures after treatment.

**Results:**

Our results are focused mainly on data obtained from neurochemical studies, demonstrating that 1MeTIQ inhibited the MK-801-induced reduction in dopamine levels in the frontal cortex and increased the 5-HT concentration. The behavioral tests revealed that acute administration of MK-801 caused disturbances in both the FM and SIT tests, while neither 1MeTIQ nor olanzapine reversed these deficits.

**Conclusion:**

1MeTIQ, although pharmacologically effective (i.e., it reverses MK-801-induced changes in monoamine activity), did not influence MK-801-induced social and cognitive deficits. Thus, our FM tests and SIT did not support the main pharmacological hypotheses that focus on dopamine system stabilization and dopamine–serotonin system interactions as probable mechanisms for inhibiting the negative symptoms of schizophrenia.

## Introduction

Schizophrenia is a devastating mental illness that occurs in 1% of the population worldwide and may be characterized by emotional impairment, cognitive deficits, and social dysfunction [1]. According to cognitive deficits, abnormalities in associative memory processes may be observed in schizophrenic subjects [2].

Fear memory (FM) is crucial for predicting and avoiding aversive and dangerous events. Thus, appropriately functioning fear promotes survival and minimizes exposure to danger [3]. FM may be assessed by using Pavlovian fear conditioning when a conditional stimulus (CS), e.g., a tone, is followed by an unconditional stimulus (US), e.g., a foot shock in a specific training context. Thereby, animals develop a fear of both the tone and the training context, and the memory of that fear is measured by assessing freezing time, which is a natural, adaptive and species-specific reaction to threat [4, 5]. Freezing behavior during CS presentation is taken as a measure of fear. The adaptive reaction of FM is disturbed in schizophrenia.

Social deficits are considered core negative symptoms in schizophrenia and are often the earliest symptoms of the illness [6, 7]. To study social withdrawal, as a schizophrenia-related symptom, the social interaction test (SIT) is used as a screening tool after drug administration [8].

MK-801 is an NMDA receptor antagonist and is commonly used in animal models to mimic schizophrenia-like behaviors [9]. MK-801 is reported to disturb working memory, memory consolidation, social interaction, contextual fear conditioning and prepulse inhibition [10–15].

Olanzapine is an atypical antipsychotic drug that is widely used in the treatment of schizophrenia [16]. Olanzapine affects many receptors, including the dopamine D2 receptor; serotonin 5-HT2A, 5-HT2C, and 5-HT6 receptors; adrenaline α1; histamine H1; and muscarine M1–M5 [17].

1-Methyl-1,2,3,4-tetrahydroisoquinoline (1MeTIQ) is an endogenous compound present in the mammalian brain, mainly in dopaminergic structures [18]. The bulk of evidence has shown that 1MeTIQ has neuroprotective [19–22], antiaddictive [23] and anxiolytic-like properties [24]. As shown in previous studies, 1MeTIQ acts as a reversible inhibitor of monoamine oxidase (MAO); therefore, it is considered an antioxidative agent [22]. Early studies on tetrahydroisoquinolines indicated that they have neuroleptic-like properties [25], and our experiments confirmed that 1MeTIQ acts as a specific antagonist of agonistic conformations of dopamine receptors and may act as an inhibitory regulator that counteracts excessive stimulation of catecholaminergic systems [20, 26, 27].

Antipsychotic drugs show therapeutic efficacy in treating positive symptoms (e.g., hallucinations) of schizophrenia, but their effectiveness in treating negative symptoms and cognitive manifestations of the illness is limited [28]. Our earlier study showed that 1MeTIQ exhibits anxiolytic and procognitive properties in an animal model of schizophrenia [24, 29]; therefore, we decided to verify whether 1MeTIQ could eliminate negative symptoms or improve memory.

The aim of the present study was to evaluate the effect of 1MeTIQ on neurochemical changes in monoamine levels within central nervous system components (specifically, the hippocampus and frontal cortex (Fcx) in a MK-801-induced model of schizophrenia. The results obtained after administration of 1MeTIQ were compared to those of olanzapine. Contextual fear conditioning was used to assess disturbances in FM, and sociability was measured in the SIT.

## Materials and methods

### Animals and treatments

All experimental procedures were approved by the Committee for Laboratory Animal Welfare and the Ethics Committee of the Institute of Pharmacology, PAS, in Krakow.

All experiments were conducted on male Sprague–Dawley rats with an initial body weight of 225–250 g. The animals were kept in standard polyacrylic cages (5 animals/cage) with free access to water and standard laboratory food. Animals were kept at room temperature (22 °C) under an artificial light/dark cycle (12/12 h, light on at 7:00). A single injection of MK-801 (0.3 mg/kg, ip in the FM test; 0.1 mg/kg, sc in the SIT) was given to induce behavioral disorders. 1MeTIQ (25 mg/kg, ip) was given as a single dose or chronically for 7 consecutive days. Olanzapine (3 mg/kg, ip) was administered once. In the combined groups, 1MeTIQ or olanzapine was administered 20 min before MK-801 injection. The last dose of 1MeTIQ in chronic treatment was given on the day of the behavioral test. Control rats were treated with vehicle (0.9% NaCl). Doses of the drugs were based on our previous experience (1MeTIQ) or the literature (MK-801 and olanzapine). Animals were divided into eight groups depending on the treatment they received (Table 1). The number of individuals was 8–10 per group. A total of 144 animals were used in the experiments.

**Table 1:** Treatments and drug doses applied to experimental groups

Treatment	SAL	MK-801 0.3 mg/0.1 mg	Olanzapine 3 mg	1MeTIQ 1x 25 mg	1MeTIQ 7x 25 mg
Group					
Control	+				
MK-801	+	+			
Olanzapine	+		+		
1MeTIQ-1x	+			+	
1MeTIQ-7x	+				+
Olanzapine + MK-801		+	+		
1MeTIQ-1x + MK-801		+		+	
1MeTIQ-7x + MK-801		+			+

**+ **refers to administration of a particular drug. Animals were divided into 8 groups, including the control (saline) group. *N* = 8–10. The doses of MK-801 were different for FM (0.3 mg/kg, *ip*) and the SIT (0.1 mg/kg, *sc*)

### Drugs

1-Methyl-1,2,3,4-tetrahydroisoquinoline (1MeTIQ) was synthesized by the Department of Drug Chemistry, Maj Institute of Pharmacology Polish Academy of Sciences, Krakow, Poland. The purity of the compound was verified by measurement of the melting point, and homogeneity was assessed on a chromatographic column. MK-801 (Sigma-Aldrich, USA) and 1MeTIQ were dissolved in sterile 0.9% NaCl solution and injected in a volume of 1 ml/kg. Olanzapine (Sigma-Aldrich, USA) was suspended in a 1% aqueous solution of Tween 80.

### Biochemical analysis of monoamines and their metabolites

Immediately after the behavioral test, rats were decapitated. The Fcx and hippocampus were dissected and frozen on solid CO_2_ (− 70 °C) and stored until biochemical assays. Dopamine (DA) along with its metabolites 3,4-dihydroxyphenylacetic acid (DOPAC), 3-methoxytyramine (3-MT) and homovanillic acid (HVA, the final metabolite); serotonin (5-HT) along with its metabolite 5-hydroxyindoleacetic acid (5-HIAA); and noradrenaline (NA) along with its metabolite normetanephrine (NM) were assayed by means of high-performance liquid chromatography (HPLC) with electrochemical detection. The chromatograph (HP 1050; Hewlett-Packard, Golden, CO, USA) was equipped with C18 columns. The sample preparation procedure was based on our previous protocol [24].

### Fear conditioning

Fear conditioning (FC) and memory tests were performed and analyzed using a computer-controlled FC system (TSE, Bad Homburg, Germany), as previously described by Chocyk et al. [3]. Each FC unit consisted of sound-attenuating housing with a loudspeaker, camera, ventilation fan and four symmetrically mounted lamps in the ceiling construction and test box.

During the experimental procedure, the animals were tested in two different arenas and contexts (A and B). For the first context (Context A), the arena (46 × 46 × 47 cm) was made of transparent acrylic and had a floor made up of stainless steel rods (4 mm in diameter) spaced 8.9 mm apart (center to center). The floor was connected to a shocker-scrambler unit for delivering shocks of defined duration and intensity. The test arena was cleaned with 1% acetic acid solution. A ventilation fan provided background noise (65 dB), and lamps provided uniform illumination of 60 lx inside the FC housing. During tests in Context A, the room lights remained on. Animals were transported to this context with transparent plastic boxes. Experimenters wore white clothes and gloves.

For the second context (Context B), the arena (46 × 46 × 47 cm) was made of black acrylic with a gray plastic floor. The arena was cleaned with 70% ethanol solution and faintly illuminated (4 lx). The tests in Context B were conducted with the room light off. Animals were transported to this context with black plastic boxes. Experimenters wore blue clothes and gloves. All sessions were recorded and archived for further verification.

FC and memory were assessed using the Pavlovian paradigm. On day 1 of the experiment, all animals were subjected to FC in Context A (acquisition/training). 1MeTIQ (25 mg/kg, ip) or olanzapine (3 mg/kg, ip) was given 35 min before training, whereas MK-801 (0.3 mg/kg, ip) was administered once, 15 min before training. In the chronic treatment groups, the last dose of 1MeTIQ was given 35 min before training. Animals were placed in context A and allowed to habituate for 180 s. Next, the animals received five tone-shock pairings in which the tone (amplitude: 80 dB; frequency: 2 kHz; duration: 10 s) coterminated with foot shock (intensity: 1 mA; duration: 1 s). The intertrial interval was 60 s. Animals were removed from context A 60 s after the last trial.

On day 2, all animals were once again exposed to Context A and were left undisturbed for 6 min (expression of contextual fear conditioning, CFC) and then returned to their home cages. Two hours later, animals were placed in a new context (Context B) and, after 180 s of habituation, received five presentations of tone alone with 61 s intertrial intervals (expression of auditory fear conditioning, AFC).

**Fig. 1:**
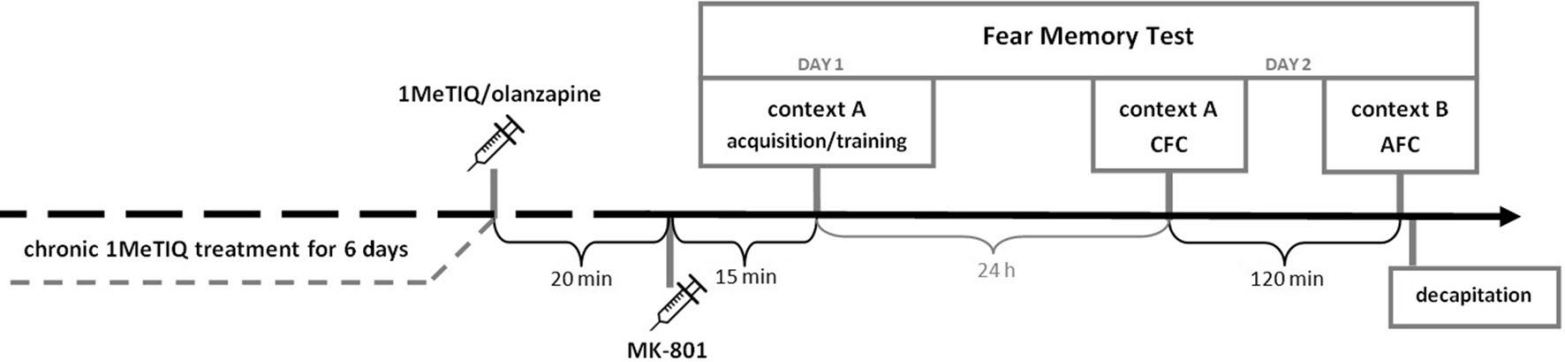
Schematic representation of the FM experiment. Chronic treatment with 1MeTIQ lasted for 7 days, and the last dose was given on the day of the experiment 20 min before MK-801. The FM test consisted of the acquisition/training phase in Context A (day 1) and the testing phase in Context A—CFC and Context B—AFC (day 2). The interval between CFC and AFC on day 2 lasted 2 h. *N* = 8–10 rats per group. After behavioral testing, animals were immediately decapitated

Behavioral responses during all sessions were recorded and automatically analyzed using FC software (TSE, Bad Homburg, Germany). Freezing (i.e., immobility) was taken as the behavioral measure of fear and was defined as the absence of all nonrespiratory movements for at least 2 s. The cumulative duration of freezing was calculated for each session and expressed as a percentage of the entire session time, excluding habituation time, in the case of AFC expression (Fig. 1).

### Social interaction test

**Fig. 2:**
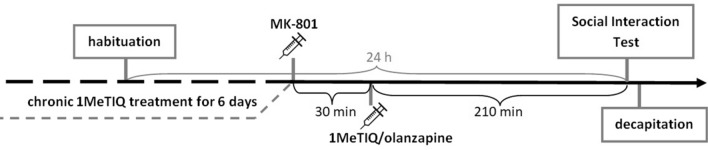
Schematic representation of the SIT. Chronic treatment with 1MeTIQ lasted for 7 days, and the last dose was given on the day of the experiment 30 min after MK-801 and 210 min before behavioral testing. The SIT consisted of habituation and general testing with a 24-h intervening interval. *N* = 8–10 rats per group. Immediately after the test, animals were decapitated

The SIT was performed using a black wooden box (60 × 60 × 25 cm). The arena was dimly illuminated with indirect light of 18 lx. Each social interaction experiment involving two rats was carried out during the light phase of the light/dark cycle. The rats were selected from separate housing cages to make a pair for the study. The paired rats were matched for body weight within 15 g. Each trial involved two same-treated rats. MK-801 (0.1 mg/kg, *sc*) was administered once, 240 min before SIT. In the combined groups 1MeTIQ (25 mg/kg, *ip*) or olanzapine (3 mg/kg, *ip*) was given 210 min after MK-801 injections. In the chronic treatment groups, the last dose of 1MeTIQ was given 30 min before SIT (Fig. 2).

Each pair of rats was diagonally placed in opposite corners of the box. The behavior of animals was measured over a 10-min period. The test box was wiped clean between each trial. Social interaction between two rats was expressed as the total time spent in social behavior, such as sniffing, genital investigation, chasing and fighting with each other. The number of episodes was counted as a separate paradigm. Each group consisted of 12 animals (6 pairs) [30].

### Statistical analysis

The results of behavioral tests and biochemical analysis (acute treatment) were analyzed by means of one-way ANOVA followed, when appropriate, by a post hoc Duncan’s multiple range test (MRT). The results from behavioral tests and biochemical analysis of chronic treatment were analyzed by means of two-way ANOVA followed, when appropriate, by a post hoc Duncan’s MRT. The results were considered statistically significant when *p* < 0.05.

## Results

### Frontal cortex—biochemical analysis; acute 1MeTIQ treatment

#### Dopamine and its metabolites

One-way ANOVA revealed a significant (*F*_5,36_ = 11.31, *p* < 0.001) effect of the applied treatment on DA levels in the Fcx of rats. Post hoc tests showed a significantly decreased DA level in animals treated with MK-801 compared to saline (*p* < 0.001), and this effect was reversed by an acute dose of either 1MeTIQ or olanzapine. In both combined groups, the DA concentration returned to the control level (Table 2).

**Table 2:** Results from the biochemical assay after acute 1MeTIQ treatment

Treatment	*n*	DA	DOPAC	3-MT	HVA
FCX					
Control	5	510 ± 29	159 ± 7	14 ± 1	155 ± 14
MK-801	5	213 ± 36***	158 ± 10	17 ± 2	121 ± 16
1MeTIQ	8	458 ± 39	96 ± 6	27 ± 5	152 ± 7
Olanzapine	10	319 ± 26**	326 ± 14***	30 ± 11	272 ± 20***
1MeTIQ + MK-801	5	490 ± 46^###^	92 ± 8*^#^	24 ± 4	127 ± 6
Olanzapine + MK-801	9	543 ± 37^###^	370 ± 36***^###^	19 ± 2	245 ± 23**^###^
*F*		F_(5/36)_ = 11.31 p < 0.001	F_(5/38)_ = 37.92 p < 0.001	F_(5/39)_ = 0.97 p = 0.45	F_(5/39)_ = 12.72 p < 0.001
HIP					
Control	5	25 ± 1	11 ± 1	15 ± 2	17 ± 2
MK-801	6	23 ± 3	17 ± 1*	13 ± 1	21 ± 3
1MeTIQ	10	34 ± 2*	12 ± 1	20 ± 3	26 ± 3
Olanzapine	9	31 ± 2	26 ± 2***	22 ± 3*	26 ± 3
1MeTIQ + MK-801	6	29 ± 2	12 ± 1^+^	10 ± 1	19 ± 3
Olanzapine + MK-801	10	22 ± 2	17 ± 2*	11 ± 1	26 ± 3
*F*		F_(5/40)_ = 4.38 p < 0.01	F_(5/39)_ = 15.42 p < 0.001	F_(5/38)_ = 4.36 p < 0.01	F_(5/38)_ = 1.51 p = 0.21

DA and its metabolites were measured in the Fcx and hippocampus using HPLC. The data were analyzed using one-way ANOVA and a post hoc Duncan’s MRT. The results are shown as the means ± SEM. *N* = 8–10 rats per group

*p < 0.05; **p < 0.01; ***p < 0.001 indicate significant changes compared to the control; ^#^*p* < 0.05; ^##^*p* < 0.01; ^###^*p* < 0.001 indicate significant changes compared to the model (MK-801) group

One-way ANOVA showed a significant (*F*_5,38_ = 37.92, *p* < 0.001) effect of the treatment on frontal DOPAC levels. Duncan’s MRT showed a decreased DOPAC level after treatment with both 1MeTIQ and MK-801 (compared to saline or MK-801; both *p* < 0.05). We observed an increased DOPAC level after olanzapine was given either alone (compared to saline; *p* < 0.001) or combined with MK-801 (compared to the saline and MK-801 groups; both *p* < 0.001) (Table 2).

One-way ANOVA revealed no significant difference (*F*_5,39_ = 0.97, *p* = 0.45) effect of the treatment on the 3-MT level (Table 2).

The same analysis showed a significant (*F*_5,39_ = 12.72, *p* < 0.001) effect of the treatment on HVA levels in the Fcx. Post hoc analysis showed a significantly increased level of HVA after olanzapine treatment compared to saline (*p* < 0.001). Olanzapine given with MK-801 increased the HVA level compared to both saline (*p* < 0.01) and MK-801 (*p* < 0.001) (Table 2).

#### Noradrenaline and normetanephrine (NM)

One-way ANOVA showed a significant (*F*_5,39_ = 4.38, *p* < 0.01) effect of the applied treatment on NA levels. Post hoc tests revealed that treatment with MK-801, 1MeTIQ or olanzapine alone caused a significant increase in the level of HVA compared to the control (*p* < 0.05; *p* < 0.001; *p* < 0.01, respectively). A similar effect was observed in groups with 1MeTIQ or olanzapine given in conjunction with MK-801 (*p* < 0.001 and *p* < 0.01, respectively) (Table 3).

**Table 3:** Results from the biochemical assay after acute 1MeTIQ treatment

Treatment	*n*	NA	NM	5-HT	5-HIAA
FCX					
Control	5	265 ± 15	17 ± 3	253 ± 12	243 ± 5
MK-801	5	307 ± 22*	26 ± 3	250 ± 18	241 ± 11
1MeTIQ	9	336 ± 11***	44 ± 4***	343 ± 11***	202 ± 9**
Olanzapine	10	316 ± 7**	20 ± 3	262 ± 9	254 ± 11
1MeTIQ + MK-801	6	341 ± 10***	48 ± 7***^###^	333 ± 14***^###^	171 ± 11***^###^
Olanzapine + MK-801	10	319 ± 9**	22 ± 2	252 ± 9	244 ± 7
*F*		F_(5/39)_ = 4.38 p < 0.01	F_(5/37)_ = 12.65 p < 0.001	F_(5/42)_ = 13.92 p < 0.001	F_(5/41)_ = 10.40 p < 0.001
HIP					
Control	6	301 ± 20	24 ± 3	226 ± 13	309 ± 12
MK-801	6	295 ± 16	25 ± 4	202 ± 18	327 ± 16
1MeTIQ	10	341 ± 20	44 ± 4***	284 ± 14**	288 ± 10
Olanzapine	9	289 ± 10	33 ± 3	235 ± 10	356 ± 9
1MeTIQ + MK-801	6	295 ± 14	32 ± 3	265 ± 10*^##^	255 ± 22^#^
Olanzapine + MK-801	9	313 ± 21	22 ± 1	217 ± 8	302 ± 23
*F*		F_*(5/40)*_ = 1.35 p = 0.26	F_(5/39)_ = 7.07 p < 0.001	F_(5/40)_ = 6.33 p < 0.001	F_(5/40)_ = 3.91 p < 0.01

NA, 5-HT and their metabolites (NM, 5-HIAA) were measured in the Fcx and hippocampus using HPLC. The data were analyzed using one-way ANOVA and a post hoc Duncan’s MRT. The results are shown as the means ± SEM. *N* = 8–10 rats per group

*p < 0.05; **p < 0.01; ***p < 0.001 indicate significant changes compared to the control; ^#^*p* < 0.05; ^##^*p* < 0.01; ^###^*p* < 0.001 indicate significant changes compared to the model (MK-801) group

One-way ANOVA revealed a significant (*F*_5,37_ = 12.65, *p* < 0.001) effect of the treatment on NM levels. 1MeTIQ caused a significant increase in the level of NM compared to the control (*p* < 0.001). 1MeTIQ given together with MK-801 increased the NM level significantly compared to those of the saline and MK-801 groups (*p* < 0.001) (Table 3).

#### Serotonin and 5-hydroxyindoleacetic acid (5-HIAA)

One-way ANOVA showed a significant (*F*_5,42_ = 13.92, *p* < 0.001) effect of the treatment on frontal 5-HT levels. Post hoc tests showed a significant increase in 5-HT after 1MeTIQ (*p* < 0.001, compared to saline) and 1MeTIQ given with MK-801 (compared to saline or MK-801; both *p* < 0.001) (Table 3).

One-way ANOVA revealed a significant (*F*_5,41_ = 10.40, *p* < 0.001) effect of the applied treatment on the 5-HIAA level. Duncan’s MRT showed a decreased 5-HIAA level after 1MeTIQ given alone (compared to saline; *p* < 0.01) and 1MeTIQ given with MK-801 (compared to saline or MK-801; both *p* < 0.001) (Table 3).

### Hippocampus—biochemical analysis; acute 1MeTIQ treatment

#### Dopamine and its metabolites

One-way ANOVA showed a significant (*F*_5,40_ = 4.38, *p* < 0.01) effect of the treatment on DA levels in the hippocampus. Post hoc analysis showed a significantly higher DA level after 1MeTIQ (*p* < 0.05) compared to saline (Table 2).

The statistical analysis showed a significant (*F*_5,39_ = 15.42, *p* < 0.001) effect of the treatment on DOPAC levels. Post hoc tests showed significantly higher amounts of DOPAC after MK-801 or olanzapine treatment compared to the control (*p* < 0.05; *p* < 0.001, respectively). A similar effect was observed in the combined group treated with olanzapine and MK-801 (*p* < 0.05) (Table 2).

One-way ANOVA revealed a significant (*F*_5,38_ = 4.36, *p* < 0.01) effect of the treatment on the 3-MT level. Post hoc tests showed that olanzapine given alone caused a significant increase in 3-MT compared to saline (*p* < 0.05) (Table 2).

The same analysis showed no significant (*F*_5,38_ = 1.51, *p* = 0.21) effect of the treatment on the HVA level (Table 2).

#### Noradrenaline and normetanephrine

One-way ANOVA revealed no significant (*F*_5,40_ = 1.35, *p* = 0.26) effect of the treatment on the NA level in the hippocampus.

The same analysis showed a significant (*F*_5,39_ = 7.07, *p* < 0.001) effect of the treatment on NM levels. Post hoc tests showed significantly increased NM levels after 1MeTIQ administration compared to the control (*p* < 0.001) (Table 3).

#### Serotonin and 5-hydroxyindoleacetic acid

One-way ANOVA showed a significant (*F*_5,40_ = 6.33, *p* < 0.001) effect of the treatment on 5-HT levels in the hippocampus. Duncan’s MRT revealed significantly increased 5-HT amounts after 1MeTIQ (compared to saline: *p* < 0.01) and 1MeTIQ combined with MK-801 (compared to saline: *p* < 0.05; MK-801: *p* < 0.01) (Table 3).

The statistical analysis showed a significant (*F*_5,40_ = 3.91, *p* < 0.01) effect of the treatment on the 5-HIAA level. Post hoc tests showed that 1MeTIQ given with MK-801 caused a significant decrease in 5-HIAA compared to MK-801-treated animals (*p* < 0.05) (Table 3).

### Frontal cortex—biochemical analysis; chronic 1MeTIQ treatment

#### Dopamine and its metabolites

Two-way ANOVA revealed no significant (*F*_1,18_ = 1.28, *p* = 0.27) effect of MK-801 on frontal DA level. The same analysis showed a significant effect of chronic 1MeTIQ (*F*_1,18_ = 9.91, *p* < 0.01) and the interaction of both treatments (*F*_1,18_ = 21.10, *p* < 0.001) on DA levels. Post hoc tests revealed significantly decreased levels of DA after MK-801 treatment compared to the control (*p* < 0.01). In the combined group, chronic 1MeTIQ reversed the effect of MK-801 and increased DA levels to the control level (*p* < 0.001) (Table 4).

**Table 4:** Results from the biochemical assay after chronic (7x) 1MeTIQ treatment

Treatment	*n*	DA	DOPAC	3-MT	HVA
FCX					
Control	5	510 ± 29	159 ± 7	14 ± 1	155 ± 14
MK-801	7	213 ± 36**	158 ± 10	17 ± 2	121 ± 16
1MeTIQ-7x	5	435 ± 61	81 ± 7***	36 ± 6**	126 ± 8
1MeTIQ-7x + MK-801	5	614 ± 54^###^	94 ± 8***^###^	28 ± 4	128 ± 7
Effect of T1 Effect of T2 Interaction of T1 + T2		F_(1/18)_ = 1.28 p = 0.27 F_(1/18)_ = 9.91 p < 0.01 F_(1/18)_ = 21.10 p < 0.001	F_(1/19)_ = 0.58 p = 0.45 F_(1/19)_ = 71.72 p < 0.0011 F_(1/19)_ = 0.73 p = 0.40	F_(1/21)_ = 0.29 p = 0.59 F_(1/21)_ = 12.50 p < 0.001 F_(1/21)_ = 1.48 p = 0.24	F_(1/22)_ = 1.90 p = 0.18 F_(1/22)_ = 0.96 p = 0.34 F_(1/22)_ = 2.48 p = 0.13
HIP					
Control	5	25 ± 1	11 ± 1	15 ± 2	17 ± 2
MK-801	8	23 ± 3	17 ± 1*	13 ± 1	21 ± 3
1MeTIQ-7x	6	34 ± 2	10 ± 1	18 ± 2	29 ± 2**
1MeTIQ-7x + MK-801	4	35 ± 7^#^	10 ± 3^#^	15 ± 2	21 ± 1
Effect of T1 Effect of T2 Interaction of T1 + T2		F_(1/19)_ = 0.01 p = 0.92 F_(1/19)_ = 9.18 p < 0.01 F_(1/19)_ = 0.73 p = 0.73	F_(1/21)_ = 3.22 p = 0.09 F_(1/21)_ = 4.68 p < 0.05 F_(1/21)_ = 3.27 p = 0.09	F_(1/22)_ = 1.15 p = 0.30 F_(1/22)_ = 2.02 p = 0.17 F_(1/22)_ = 0.19 p = 0.67	F_(1/21)_ = 0.90 p = 0.35 F_(1/21)_ = 5.94 p < 0.05 F_(1/21)_ = 5.53 p < 0.05

DA and its metabolites (DOPAC. 3-MT. HVA) were measured in the Fcx and hippocampus using HPLC. The data were analyzed using two-way ANOVA and a post hoc Duncan’s MRT. The results are shown as the means ± SEM. *N* = 8–10 rats per group

*p < 0.05; **p < 0.01; ***p < 0.001 indicate significant changes compared to the control; ^#^*p* < 0.05; ^##^*p* < 0.01; ^###^*p* < 0.001 indicate significant changes compared to the model (MK-801) group

Two-way ANOVA revealed a nonsignificant (*F*_1,19_ = 0.58, *p* = 0.45) effect of MK-801 and a significant (*F*_1,19_ = 71.72, *p* < 0.001) effect of chronic 1MeTIQ on DOPAC levels. The effect of the interaction of both treatments was found to be nonsignificant (*F*_1,19_ = 0.73, *p* = 0.40). Duncan’s MRT showed decreased DOPAC levels after chronic 1MeTIQ treatment compared to the control (*p* < 0.001). A similar effect was observed in animals treated with both 1MeTIQ and MK-801 (compared to saline or MK-801: both *p* < 0.001) (Table 4).

Two-way ANOVA showed a nonsignificant (*F*_1,21_ = 0.29, *p* = 0.59) effect of MK-801 and a significant (*F*_1,21_ = 12.50, *p* < 0.01) effect of chronic 1MeTIQ on the 3-MT level. The interaction of both treatments was found to be nonsignificant (*F*_1,21_ = 1.48, *p* = 0.24). Post hoc tests showed increased 3-MT levels after chronic 1MeTIQ administration compared to the control (*p* < 0.01) (Table 4).

Two-way ANOVA showed no significant effect of MK-801 (*F*_1,22_ = 1.90, *p* = 0.18), chronic 1MeTIQ (*F*_1,22_ = 0.96, *p* = 0.34) or the interaction of both treatments (*F*_1,22_ = 2.48, *p* = 0.13) on the HVA level in the frontal cortex (Table 4).

#### Noradrenaline and normetanephrine

Two-way ANOVA showed a nonsignificant (*F*_1,21_ = 0.65, *p* = 0.43) effect of MK-801 and a significant (*F*_1,21_ = 18.44, *p* < 0.001) effect of chronic 1MeTIQ on NA levels. Two-way ANOVA showed a nonsignificant (*F*_1,21_ = 2.52, *p* = 0.13) effect of the interaction of both treatments on NA levels in the Fcx. Duncan’s MRT revealed increased NA levels after chronic 1MeTIQ, given either alone or with MK-801, compared to saline (*p* < 0.001 and *p* < 0.01, respectively) (Table 5).

**Table 5:** Results from the biochemical assay after chronic (7x) 1MeTIQ treatment

Treatment	*n*	NA	NM	5-HT	5-HIAA
FCX					
Control	5	265 ± 15	17 ± 3	253 ± 12	243 ± 8
MK-801	9	307 ± 22	26 ± 3	250 ± 18	241 ± 8
1MeTIQ-7x	5	368 ± 16***	53 ± 3***	358 ± 17***	187 ± 6***
1MeTIQ-7x + MK-801	6	354 ± 13**	42 ± 3***^###^	349 ± 15***^###^	196 ± 8***^###^
Effect of T1 Effect of T2 Interaction of T1 + T2		F_(1/21)_ = 0.65 p = 0.43 F_(1/21)_ = 18.44 p < 0.001 F_(1/21)_ = 2.52 p = 0.13	F_(1/22)_ = 0.21 p = 0.65 F_(1/22)_ = 83.58 p < 0.001 F_(1/22)_ = 11.17 p < 0.01	F_(1/23)_ = 0.11 p = 0.75 F_(1/23)_ = 38.21 p < 0.001 F_(1/23)_ = 0.03 p = 0.85	F_(1/22)_ = 0.22 p = 0.64 F_(1/22)_ = 43.59 p < 0.001 F_(1/22)_ = 0.58 p = 0.45
HIP					
Control	6	301 ± 20	24 ± 3	226 ± 13	309 ± 12
MK-801	9	295 ± 16	25 ± 4	202 ± 18	327 ± 16
1MeTIQ-7x	6	356 ± 16*	44 ± 3**	337 ± 26**	273 ± 9
1MeTIQ-7x + MK-801	6	369 ± 18*^#^	41 ± 4**^##^	317 ± 21**^##^	299 ± 32
Effect of T1 Effect of T2 Interaction of T1 + T2		F_(1/23)_ = 0.03 p = 0.85 F_(1/23)_ = 12.13 p < 0.01 F_(1/23)_ = 0.26 p = 0.62	F_(1/22)_ = 0.08 p = 0.77 F_(1/22)_ = 21.38 p < 0.001 F_(1/22)_ = 0.36 p = 0.55	F_(1/23)_ = 0.94 p = 0.34 F_(1/23)_ = 25.25 p < 0.001 p < 0.01 p = 0.93	F_(1/22)_ = 1.40 0.25p = F_(1/22)_ = 2.89 p < 0.10 F_(1/22)_ = 0.04 p = 0.83

NA, 5-HT and their metabolites (NM. 5-HIAA) were measured in the Fcx and hippocampus using HPLC. The data were analyzed using two-way ANOVA and a post hoc Duncan’s MRT. The results are shown as the means ± SEM. *N* = 8–10 rats per group

*p < 0.05; **p < 0.01; ***p < 0.001 indicate significant changes compared to the control; ^#^*p* < 0.05; ^##^*p* < 0.01; ^###^*p* < 0.001 indicate significant changes compared to the model (MK-801) group

Two-way ANOVA showed a nonsignificant (*F*_1,22_ = 0.21, *p* = 0.65) effect of MK-801 on NM level. The same analysis showed a significant effect of chronic 1MeTIQ (*F*_1,22_ = 83.58, *p* < 0.001) and the interaction of both treatments (*F*_1,22_ = 11.17, *p* < 0.01) on NM amount. Post hoc tests revealed significantly increased levels of NM in the chronic 1MeTIQ group compared to the saline group (*p* < 0.001). A similar effect was observed in the combined group compared to the control and MK-801 animals (both *p* < 0.001) (Table 5).

#### Serotonin and 5-hydroxyindoleacetic acid

Two-way ANOVA revealed a nonsignificant (*F*_1,23_ = 0.11, *p* = 0.75) effect of MK-801 and a significant (*F*_1,23_ = 38.21, *p* < 0.001) effect of chronic 1MeTIQ on the 5-HT amount. The interaction of both treatments was found to have a nonsignificant (*F*_1,23_ = 0.03*, p* = 0.85) effect on 5-HT level. Post hoc tests showed a significant increase in 5-HT after chronic 1MeTIQ, given either alone or with MK-801, compared to the 5-HT levels of control and MK-801 animals (all *p* < 0.001) (Table 5).

Two-way ANOVA revealed a nonsignificant (*F*_1,22_ = 0.22, *p* = 0.64) effect of MK-801 and a significant (*F*_1,22_ = 43.59, *p* < 0.001) effect of chronic 1MeTIQ on 5-HIAA levels. The same analysis showed a nonsignificant (*F*_1,22_ = 0.58, *p* = 0.45) effect of interaction of both treatments. Post hoc tests revealed depletion of 5-HIAA in the chronic 1MeTIQ group compared to the saline group (*p* < 0.001). A similar effect was observed in the combined group compared to the saline (*p* < 0.001) and MK-801 groups (*p* < 0.001) (Table 5).

### Hippocampus—biochemical analysis; chronic 1MeTIQ treatment

#### Dopamine and its metabolites

Two-way ANOVA revealed a nonsignificant (*F*_1,19_ = 0.01, *p* = 0.92) effect of MK-801 and a significant (*F*_1.19_ = 9.18, *p* < 0.01) effect of chronic 1MeTIQ on DA amount. The same analysis showed a nonsignificant (*F*_1,19_ = 0.73, *p* = 0.73) effect of interaction of both treatments. Duncan’s MRT revealed that chronic 1MeTIQ given together with MK-801 significantly increased DA levels compared to those of the MK-801 group (*p* < 0.05) (Table 4).

The same analysis showed a nonsignificant (*F*_1,21_ = 3.22, *p* = 0.09) effect of MK-801 and a significant (*F*_1,21_ = 4.68, *p* < 0.05) effect of chronic 1MeTIQ on DOPAC amount. The two treatments had no significant interaction effect (*F*_1,21_ = 3.27, *p* = 0.09). Post hoc tests showed a significant increase in DOPAC after MK-801 compared to the control (*p* < 0.05). Chronic 1MeTIQ given with MK-801 decreased DOPAC levels compared to MK-801 animals (*p* < 0.05) and restored DOPAC to control levels (Table 4).

Two-way ANOVA revealed a nonsignificant effect of MK-801 (*F*_1,22_ = 1.15, *p* = 0.30), chronic 1MeTIQ (*F*_1,22_ = 2.02, *p* = 0.17) or interaction of both treatments (*F*_1,22_ = 0.19, *p* = 0.67) on 3-MT level (Table 4).

Statistical analysis showed a nonsignificant (*F*_1.21_ = 0.90, *p* = 0.35) effect of MK-801, a significant effect of chronic 1MeTIQ (*F*_1,21_ = 5.94, *p* < 0.05) and a significant interaction of both treatments (*F*_1,21_ = 5.53, *p* < 0.05) on HVA amount. Post hoc tests showed a significant increase in HVA after chronic 1MeTIQ administration compared to saline (*p* < 0.01) (Table 4).

#### Noradrenaline and normetanephrine

Two-way ANOVA showed a nonsignificant (*F*_1,23_ = 0.03, *p* = 0.85) effect of MK-801 and a significant (*F*_1,23_ = 12.13, *p* < 0.01) effect of chronic 1MeTIQ on NA amount. There was a nonsignificant (*F*_1,23_ = 0.26, *p* = 0.62) effect of interaction of both treatments. Duncan’s MRT showed that chronic 1MeTIQ given alone increased NA levels compared to saline (*p* < 0.05). A similar effect was observed in the combined group compared to the control or MK-801 animals (both *p* < 0.05) (Table 5).

Two-way ANOVA showed a nonsignificant (*F*_1,22_ = 0.08, *p* = 0.77) effect of MK-801 and a significant (*F*_1,22_ = 21.38, *p* < 0.001) effect of chronic 1MeTIQ on NM levels. The same analysis showed a nonsignificant (*F*_1,22_ = 0.36, *p* = 0.55) effect of interaction of both treatments. Post hoc tests revealed an increased NM amount after chronic 1MeTIQ compared to the control (*p* < 0.01). A similar effect was observed in the group treated with both chronic 1MeTIQ and MK-801 compared to the control and MK-801 groups (both *p* < 0.01) (Table 5).

#### Serotonin and 5-hydroxyindoleacetic acid

Two-way ANOVA revealed a nonsignificant effect of MK-801 (*F*_1,23_ = 0.94, *p* = 0.34) and a nonsignificant interaction between the two treatments (*F*_1,23_ = 0.01, *p* = 0.93) on 5-HT level. However, there was a significant (*F*_1,23_ = 25.25, *p* < 0.001) effect of chronic 1MeTIQ on the 5-HT amount. Duncan’s MRT showed that chronic 1MeTIQ caused a significant increase in 5-HT compared to the control (*p* < 0.01). A similar effect was observed in the combined group when compared to the saline or MK-801 group (both *p* < 0.01) (Table 5).

Two-way ANOVA showed no significant effect of MK-801 (*F*_1,22_ = 1.40, *p* = 0.25), chronic 1MeTIQ (*F*_1,22_ = 2.89, *p* = 0.10) and the interaction of both treatments (*F*_1,22_ = 0.04, *p* = 0.83) on the 5-HIAA amount (Table 5).

### Fear conditioning

#### Acute treatment with 1MeTIQ—contextual fear conditioning (CFC)

**Fig. 3:**
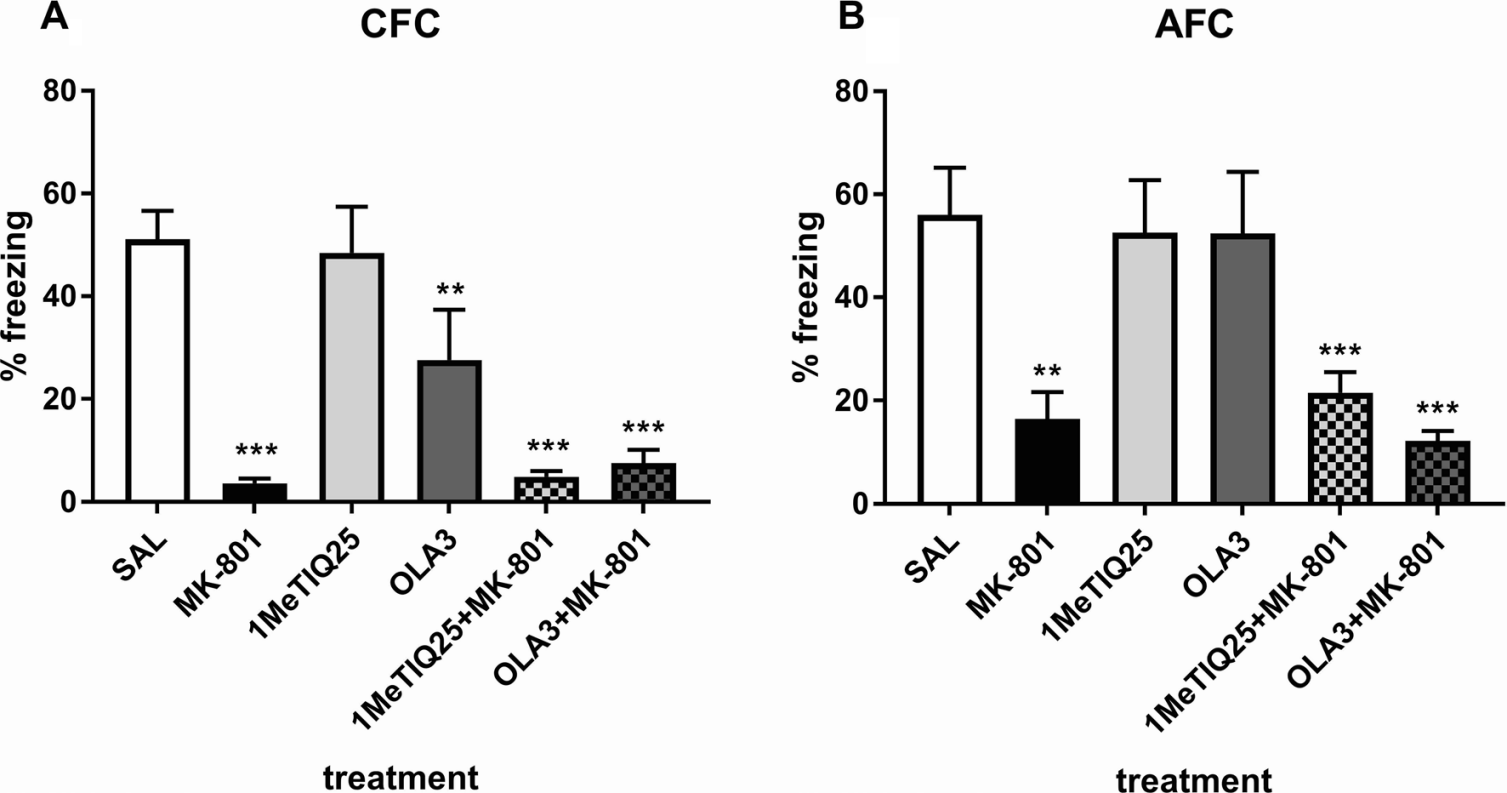
The effect of MK-801 (0.3 mg/kg, *sc*), acute 1MeTIQ (25 mg/kg, *ip*) and olanzapine (3 mg/kg, *ip*) administration on the expression of fear conditioning in the forms of CFC (**a**) and AFC **b** in the testing phase (day 2). The data were analyzed using one-way ANOVA and a post hoc Duncan’s MRT. The data are shown as the means ± SEM and expressed as a percentage of the session time. *N* = 8–10 rats per group. *p < 0.05; **p < 0.01; ***p < 0.001 compared to the control (saline) group. Significant changes were not observed when compared to the MK-801-treated group

One-way ANOVA showed a significant effect (*F*_5,46_ = 15.50, *p* < 0.001) of treatment on % freezing time in CFC. Post hoc tests showed a significant (*p* < 0.001) decrease in % freezing time after MK-801 treatment. Acute administration of olanzapine significantly (*p* < 0.01) decreased the % freezing time compared to the saline group. Combined treatment with both 1MeTIQ and MK-801 significantly (*p* < 0.001) reduced the % freezing time compared to that of the control group. Olanzapine given with MK-801 significantly (*p* < 0.001) decreased the % freezing time in the CFC (Fig. 3a).

### Acute treatment with 1MeTIQ—auditory fear conditioning (AFC)

One-way ANOVA revealed a significant effect (*F*_5,45_ = 7.14, *p* < 0.001) of applied treatment on % freezing time in AFC. Post hoc tests showed a significant (*p* < 0.01) decrease in % freezing time after MK-801 treatment. Combined treatment with both 1MeTIQ and MK-801 significantly (*p* < 0.01) decreased the % freezing time in the AFC compared to the saline group. Olanzapine given together with MK-801 significantly (*p* < 0.001) reduced % freezing time. However, neither of the combined treatments reversed the effect of MK-801 (Fig. 3b).

### Chronic treatment with 1MeTIQ—contextual fear conditioning (CFC)

**Fig. 4:**
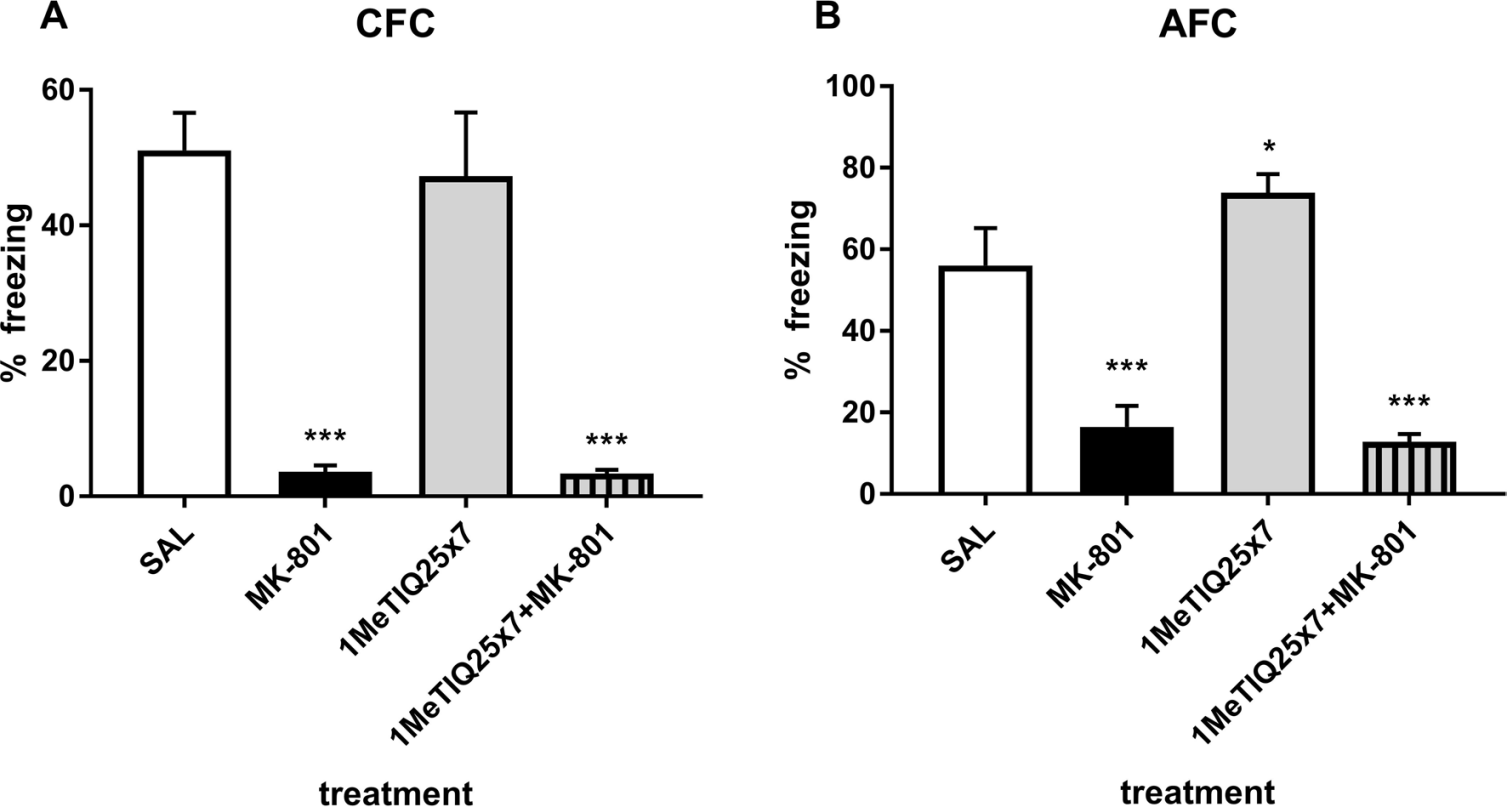
The effect of MK-801 (0.3 mg/kg, *sc*) and chronic (7x) 1MeTIQ (25 mg/kg, *ip*) administration on the expression of fear conditioning in the forms of CFC (**a**) and AFC **b** in the testing phase (day 2). The data were analyzed using two-way ANOVA and a post hoc Duncan’s MRT. The data are shown as the means ± SEM and expressed as a percentage of the session time. *N* = 8–10 rats per group. *p < 0.05; **p < 0.01; ***p < 0.001 compared to the control (SAL) group. Significant changes were not observed when compared to the MK-801-treated group

Two-way ANOVA revealed a significant effect of MK-801 (*F*_1,32_ = 87.9, *p* < 0.001) on % freezing time in the CFC. The same analysis showed no significant effect (*F*_1,32_ = 0.17, *p* = 0.68) of chronic 1MeTIQ on measured parameter. The interaction of both treatments had no significant effect (*F*_1,32_ = 0.13, *p* = 0.72) on % freezing time. Post hoc tests showed a significant (*p* < 0.001) decrease in %freezing time in the CFC after MK-801 treatment. Duncan’s MRT showed a significant (*p* < 0.001) decrease in %freezing time in the combined treatment (chronic 1MeTIQ and acute MK-801) (Fig. 4a).

### Chronic treatment with 1MeTIQ—auditory fear conditioning (AFC)

Two-way ANOVA showed a significant effect (*F*_1,31_ = 84.28, *p* < 0.001) of MK-801 on % freezing time. However, the same analysis showed no significant effect (*F*_1,31_ = 1.66, *p* = 0.21) of chronic 1MeTIQ on % freezing time in AFC. The interaction of both treatments had no significant effect (*F*_1,32_ = 3.88, *p* = 0.06) on % freezing time. Post hoc tests revealed a significant (*p* < 0.001) decrease in % freezing time in MK-801-treated animals. Chronic administration of 1MeTIQ alone produced a significant (*p* < 0.05) increase in % freezing time. Treatment with both chronic 1MeTIQ and acute MK-801 significantly (*p* < 0.001) decreased % freezing time (Fig. 4b).

### Social interaction test

#### Acute treatment with 1MeTIQ

##### Interaction time

**Fig. 5:**
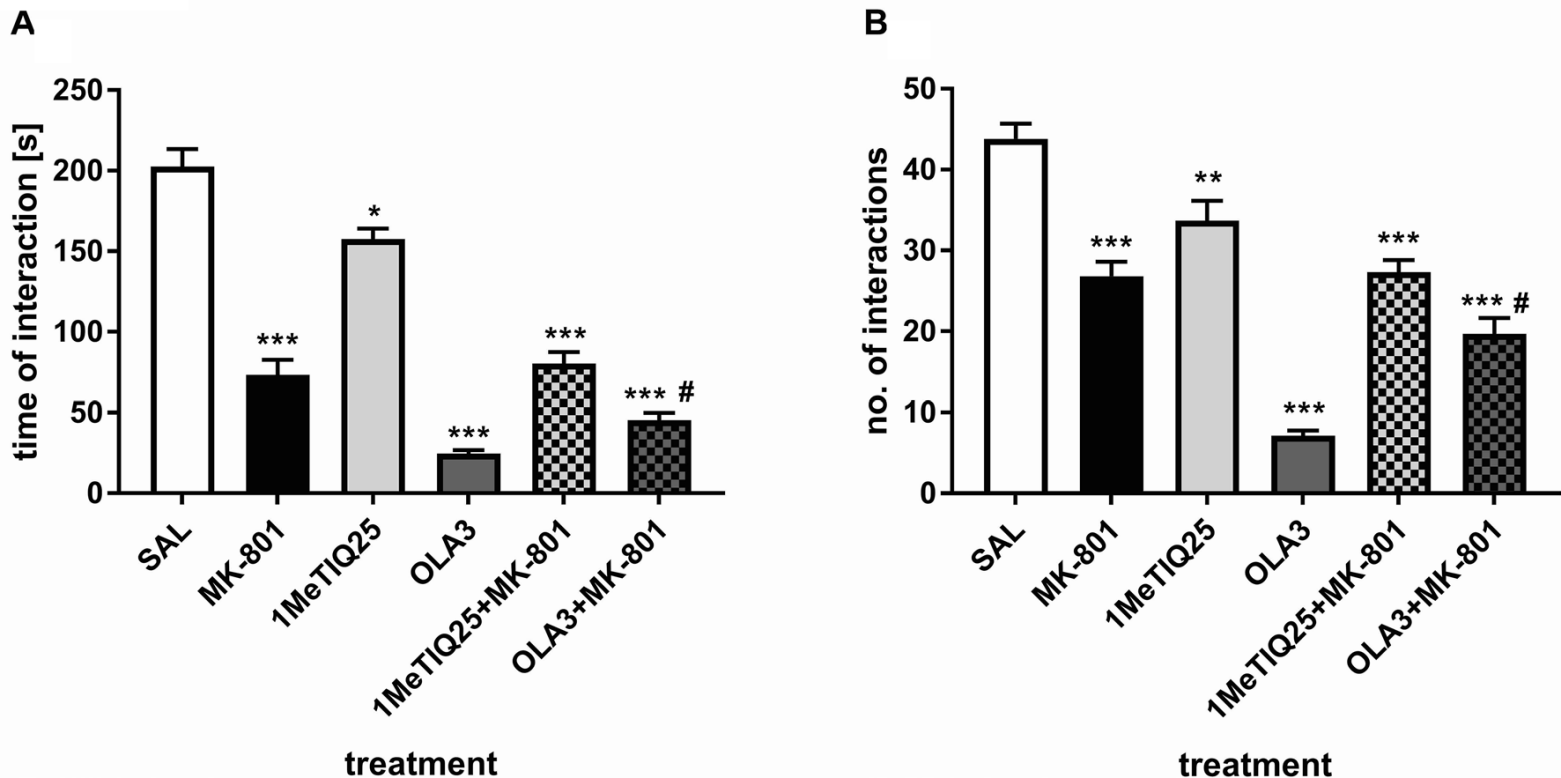
The effect of MK-801 (0.3 mg/kg, *sc*), acute 1MeTIQ (25 mg/kg, *ip*) and olanzapine (3 mg/kg, *ip*) administration on the duration of social interaction (**a**) and number of social interactions **b** in the SIT. The data were analyzed using one-way ANOVA and a post hoc Duncan’s MRT. The data are shown as the means ± SEM. *N* = 8–10 rats per group. *p < 0.05; **p < 0.01; ***p < 0.001 compared to the control (SAL) group. ^#^*p* < 0.05; ^##^*p* < 0.01; ^###^*p* < 0.001 when compared to the MK-801-treated group

One-way ANOVA revealed a significant (*F*_5,48_ = 90.77, *p* < 0.001) effect of applied treatment on the time of social interaction. Post hoc tests showed a significant (*p* < 0.001) decrease in the amount of social interaction time after MK-801 treatment. A single dose of 1MeTIQ significantly (*p* < 0.05) reduced the amount of time of social interaction when compared to saline. In olanzapine-treated animals, a strong reduction in the amount of interaction time was observed (*p* < 0.001). In animals treated with both 1MeTIQ and MK-801, the amount of social interaction time was significantly (*p* < 0.001) decreased when compared to saline. Animals treated with both olanzapine and MK-801 showed a significantly decreased amount of social interaction time compared to both saline and MK-801 animals (*p* < 0.001 and *p* < 0.05, respectively) (Fig. 5a).

##### No. of interactions

Statistical analysis showed a significant (*F*_5,48_ = 43.71, *p* < 0.001) effect of the treatment on the number of social interactions. Post hoc analysis revealed a decrease in the number of social incidents after MK-801, 1MeTIQ, or olanzapine (*p* < 0.001) administration compared to saline. A similar effect was observed in the group treated with both 1MeTIQ and MK-801. Treatment with olanzapine and MK-801 combined significantly lowered the number of social interactions compared to both the control and MK-801 groups (*p* < 0.001 and *p* < 0.05, respectively) (Fig. 5b).

#### Chronic treatment with 1MeTIQ

##### Interaction time

**Fig. 6:**
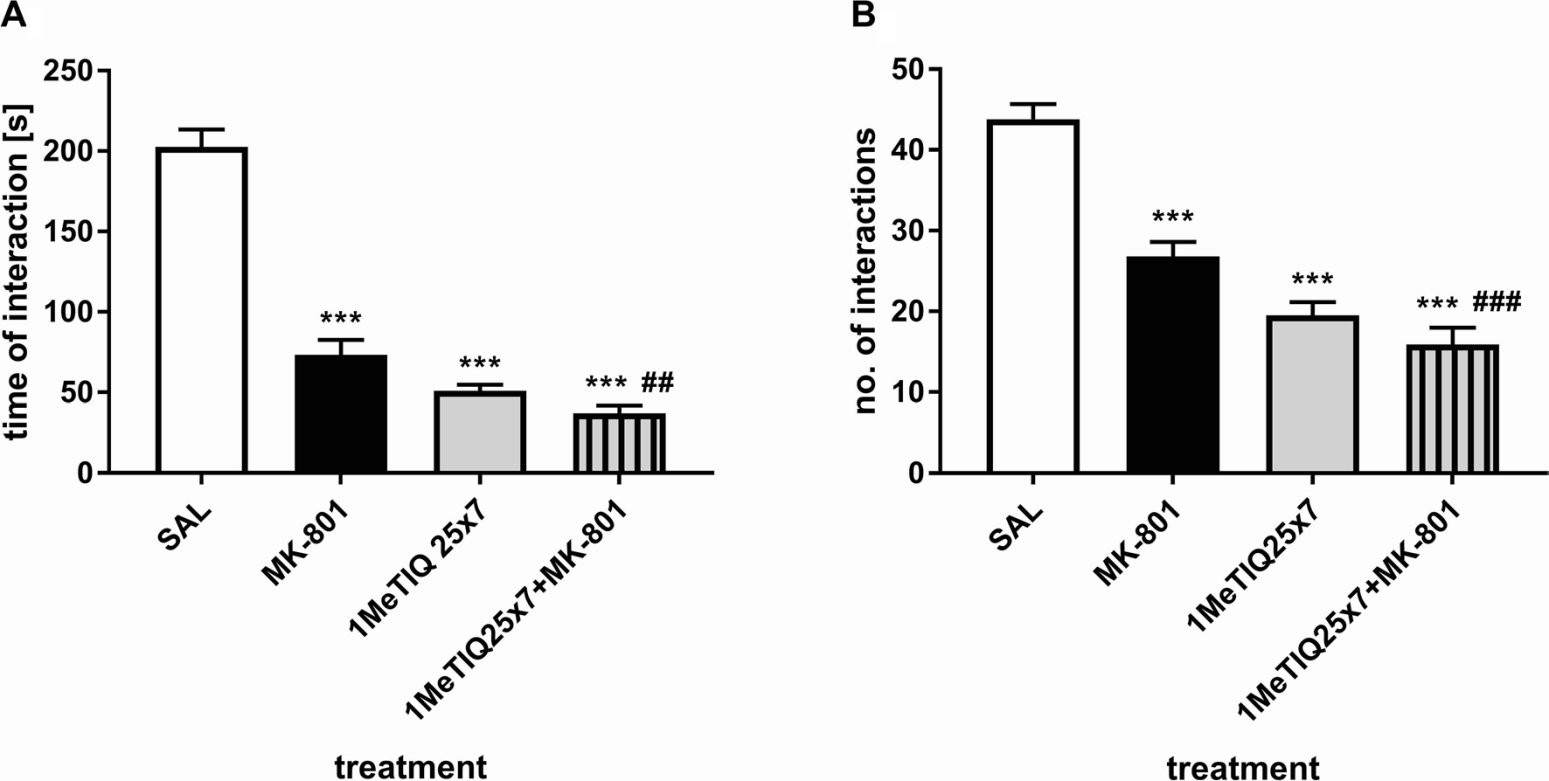
The effect of MK-801 (0.3 mg/kg, *sc*) and chronic (7x) 1MeTIQ (25 mg/kg, *ip*) administration on the duration of social interaction (**a**) and number of social interactions **b** in the SIT. The data were analyzed using two-way ANOVA and a post hoc Duncan’s MRT. The data are shown as the means ± SEM. *N* = 8–10 rats per group. *p < 0.05; **p < 0.01; ***p < 0.001 compared to the control (SAL) group. ^#^*p* < 0.05; ^##^*p* < 0.01; ^###^*p* < 0.001 when compared to the MK-801-treated group

Two-way ANOVA showed a significant effect of MK-801 (*F*_1,17_ = 58.40, *p* < 0.001), chronic 1MeTIQ *(F*_1,17_ = 100.77, *p* < 0.001) or the interaction of both treatments (*F*_1,17_ = 38.04, *p* < 0.001) on the time of social interaction. Post hoc tests revealed a significant decrease in the amount of interaction time (*p* < 0.001) after MK-801 or chronic 1MeTIQ administration. In the combined group, chronic 1MeTIQ given together with MK-801 caused a significant decrease in the amount of social interaction time when compared to both control (*p* < 0.001) and MK-801-treated animals (*p* < 0.05) (Fig. 6a).

##### No. of interactions

Two-way ANOVA showed a significant effect of MK-801 (*F*_1,17_ = 17.62, *p* < 0.001), chronic 1MeTIQ (*F*_1,17_ = 51.71, *p* < 0.001) or the interaction of both treatments (*F*_1,17_ = 7.44, *p* < 0.05) on the number of social interactions in the behavioral test. Duncan’s MRT revealed a significant decrease in the number of social interactions after MK-801 treatment or chronic 1MeTIQ treatment (both *p* < 0.001). Chronic 1MeTIQ administered together with MK-801 caused a significant depletion of social interactions compared to both the saline (*p* < 0.001) and MK-801 (*p* < 0.01) groups (Fig. 6b).

## Discussion

The main point of the presented results was connected with data obtained in neurochemical studies and demonstrated that 1MeTIQ inhibits the MK-801-induced reduction in DA levels in the Fcx and increases 5-HT concentration. There is evidence that acute administration of NMDA receptor antagonists, such as MK-801 or ketamine, increases DA release in the Fcx and striatum [31, 32]. Our previous in vivo microdialysis study confirmed an increase in DA release in the Fcx caused by systemic injection of MK-801 [33], but in the present ex vivo study, we observed a significant decrease in the tissue concentration of DA, especially in the Fcx, after MK-801 administration, perhaps suggesting its release to the extracellular space. Interestingly, this effect was reversed by both single and multiple administrations of 1MeTIQ; DA levels were restored to just above the control level (Tables 2, 4). An enhancement of DAergic and NAergic transmission in the Fcx can effectively reverse impaired cognition and flexibility in schizophrenia. Therefore, systemic administration of mazindol (an inhibitor of DA and NA reuptake) showed beneficial effects on deficits in the attentional set-shifting task [34]. Some results have indicated that D2 receptors are involved in working memory, and their activation leads to protection against social withdrawal [35, 36]. Olanzapine acts as a D2 receptor antagonist. Some authors point out that DA receptor antagonists such as haloperidol or clozapine significantly reduce locomotor activity [37, 38], and such action may mask any potential inhibitory effects of these compounds on FM. Both 1MeTIQ and olanzapine also reduced the locomotor activity of rats. If the animals have reduced motor skills, the number and duration of social contacts is automatically reduced. Therefore, based on these tests, we are not able to determine whether the tested compounds reverse MK-801-induced social and cognitive deficits. Our results confirm data from other authors that olanzapine did not antagonize MK-801-induced deficit

s in social interaction [39]. The mechanism of action of olanzapine is very unclear, and some authors emphasize that olanzapine counteracts the decrease in BDNF levels caused by MK-801 [40]. A similar effect was observed in patients with schizophrenia [41]. However, other studies show no effect of olanzapine on BDNF levels [42, 43].

1MeTIQ acts as a partial agonist of DA receptors [20]. In addition, 1MeTIQ possesses a low affinity for NMDA receptors and binds to the glycine site [19]. The present results indicated that a single injection of 1MeTIQ does not interfere with the social interaction of rats. When given together with MK-801, it causes a synergistic effect leading to larger deficits. Perhaps this effect is due to the influence of both compounds on NAergic transmission in the limbic area, especially the Fcx (Table 3). It is important to mention that dysregulation of the NAergic system may play an important role in both the negative and positive symptoms of schizophrenia. It was observed in clinical studies that NA was elevated in the blood plasma as well as in the cerebrospinal fluid of patients with schizophrenia [44, 45]. Interestingly, much research evidence indicates the essential participation of NA and its receptors (α1, α2, β) in memory function and social interactions, which may play opposite roles in these activities [46, 47].

Other data suggest that an important contribution to the action of antipsychotic drugs on cognitive symptoms of schizophrenia is the balance in activity between the 5-HT1A and DA D2 receptors [48]. MK-801-induced deficits in recognition memory and SIT are associated with the 5-HT1A receptor [49, 50]. 1MeTIQ (given alone or combined with MK-801) significantly increased the concentration of 5-HT in the Fcx and hippocampus (Tables 3, 5). For that reason, we expected a therapeutic effect of 1MeTIQ in these behavioral tests. As we demonstrated earlier, an acute dose of 1MeTIQ decreased locomotor activity in rats and completely inhibited the locomotor hyperactivity induced by MK-801 [33]. 1MeTIQ antagonized some neurochemical and behavioral effects of MK-801, but it did not reverse the behavioral disorders caused by MK-801 in the FM and SIT tests. Similarly, as has been previously demonstrated, 1MeTIQ did not improve sensorimotor gating deficits induced by MK-801 [33].

In this study, we set out to determine the effects of 1MeTIQ on FM and social recognition deficits induced by the noncompetitive NMDA receptor antagonist MK-801. It is well documented that hypoactivation of the glutamatergic system is a key mechanism underlying schizophrenia, and NMDA receptor involvement has been confirmed for FM [51] and social activity in rats [12, 39]. Fear conditioning provides an elementary form of learning through which animals learn to predict an aversive stimulus and learn to react appropriately to a threat [51], while deficits measured in the SIT mimic negative symptoms of schizophrenia in rats.

In our study, acute administration of MK-801 disturbed both FM and social interaction (Figs. 3, 4, 5, 6), which is consistent with the results obtained by other authors [39, 52, 53].

The results obtained in the FM test indicated that a single 1MeTIQ administration does not cause deficits in the fear conditioning test for either the CFC or AFC (Fig. 3a, b). At the same time, in the combined group, 1MeTIQ did not reverse the effects caused by MK-801 administration (Fig. 3a, b). Olanzapine given alone produced deficits in the CFC (Fig. 3a), and when given in conjunction with MK-801, it did not inhibit MK-801-induced deficits in either CFC and AFC (Fig. 3a, b). There are discrepancies in the literature regarding the effects of olanzapine: Siemiątkowski et al. [54] indicated that acute treatment with olanzapine decreased preshock contextual vocalizations and tended to diminish postshock vocalization; Milstein et al. [55] showed that chronic treatment with olanzapine caused specific deficits in extinction of fear conditioning and working memory, while Mead et al. [56] reported that olanzapine alleviates a variety of fear-related responses. Inoue and coworkers [57] found that olanzapine and clozapine reduced contextual fear in a dose-dependent manner. It seems that such different effects of olanzapine are caused by different protocols and doses of administration. Interestingly, chronic administration of 1MeTIQ did not change the behavior of the rats in the CFC but significantly (*p* < 0.05) increased freezing in the AFC among treated rats compared to the control group (Fig. 4b). There is evidence that elevated levels of synaptic DA result in reduced CFC (see review [58]). For this reason, different dopaminergic drugs have been examined for FM: bupropion (a DA and NA reuptake inhibitor) and aripiprazole (a partial D2 and 5-HT1A receptor agonist) reduce the retrieval of contextual fear; in contrast, haloperidol has no effect [57, 59, 60].

The effect of 1MeTIQ and olanzapine was also demonstrated in another behavioral test—the SIT. Our knowledge of the neural mechanisms underlying social behavior is limited. Pharmacological modulation of social interaction has revealed that psychomotor stimulants such as amphetamine and methylphenidate profoundly inhibit social play through a NAergic mechanism of action in the Fcx [61–64]. More recently, Achterberg et al. [65] investigated the different effects of DA and NA in social play in rats, determining that DA was more strongly associated with motivation for play, while enhanced NA negatively modulated both motivation and expression of social play. Furthermore, it was determined that infusion of methylphenidate and atomoxetine (NA reuptake inhibitors) into the prefrontal and limbic brain areas inhibited social behavior via increased NAergic activity [64]. In contrast, Kohli and coworkers demonstrated in a microdialysis study that oxytocin promotes social behavior by selectively elevating DA overflow in the nucleus accumbens but not in the Fcx [66]. Our study showed that an acute dose of 1MeTIQ slightly reduced (*p* < 0.05) both the number and duration of social interactions (Fig. 5a, b), and in the same study, 1MeTIQ did not reverse the social withdrawal induced by a single dose of MK-801. In the same experiment, a single dose of olanzapine strongly reduced both the number and duration of social interactions (*p* < 0.001), whereas olanzapine coadministered with MK-801 potentiated NMDA antagonist-induced disorder (Fig. 5a, b). Moreover, chronic administration of 1MeTIQ (for 7 days) induced social recognition deficits (*p* < 0.001) similar to those caused by a single administration of MK-801 (Fig. 6a, b). Multiple 1MeTIQ injections combined with a single dose of MK-801 increased NMDA antagonist-induced disorders (Fig. 6a, b). The results above revealed that neither 1MeTIQ nor olanzapine reversed MK-801-induced deficits in social interaction in rats. Atypical antipsychotics are considered effective in treating positive, negative, and cognitive symptoms, but treatment efficacy varies across the different pharmacological agents [67–70]. For example, treatment with the atypical neuroleptic olanzapine or risperidone did not reverse negative symptoms of schizophrenia either in the clinic [71] or in an animal model: MK-801-induced deficits in social recognition in rats [39]. We must remember that preclinical studies are conducted in animal models. All animal models are imperfect and reflect human diseases only to a limited extent. This applies in particular to mental illnesses, which are very difficult to model, e.g., depression or schizophrenia.

## Conclusion

Our neurochemical studies showed that 1MeTIQ completely antagonized the MK-801-induced reduction in DA levels in the Fcx and significantly increased the 5-HT concentration in both investigated structures. On the other hand, the behavioral results demonstrated that neither 1MeTIQ nor olanzapine antagonized negative symptoms (as shown by the FM and SIT) in an MK-801-induced model of schizophrenia. Therefore, we would like to investigate the essential role of NA in the Fcx in deepening the negative symptoms of schizophrenia. Moreover, the main pharmacological hypotheses focusing on DA system stabilization and DA-serotonin system interactions as the probable mechanism ameliorating negative symptoms of schizophrenia were not fully confirmed in the behavioral tests (FM and SIT) that were used.

## Abbreviations

1MeTIQ: 1-Methyl-1,2,3,4-tetrahydroisoquinoline
3-MT: 3-Methoxytyramine
5-HIAA: 5-Hydroxyindoleacetic acid
5-HT: Serotonin
AFC: Auditory fear conditioning
CFC: Contextual fear conditioning
COMT: Catechol-O-methyltransferase
DOPAC: 3,4-Dihydroxyphenylacetic acid
Fcx: Frontal cortex
FM: Fear memory
HPLC: High-performance liquid chromatography
HVA: Homovanillic acid
MAO: Monoamine oxidase
NM: Normetanephrine
NMDA: *N*-Methyl-d-aspartate
SIT: Social interaction test
